# Antiviral Polymers: A Review

**DOI:** 10.3390/polym14091634

**Published:** 2022-04-19

**Authors:** Ali Akbari, Ashkan Bigham, Vahid Rahimkhoei, Sina Sharifi, Esmaiel Jabbari

**Affiliations:** 1 Solid Tumor Research Center, Research Institute for Cellular and Molecular Medicine, Urmia University of Medical Sciences, Urmia 57147, Iran; akbari.a@umsu.ac.ir (A.A.); vahidrahimkhoei@gmail.com (V.R.); 2 Institute of Polymers, Composites and Biomaterials—National Research Council (IPCB-CNR), Viale J.F. Kennedy 54—Mostra d’Oltremare Pad. 20, 80125 Naples, Italy; ashkanbigham@gmail.com; 3 Disruptive Technology Laboratory, Massachusetts Eye and Ear and Schepens Eye Research Institute, Department of Ophthalmology, Harvard Medical School, Boston, MA 02115, USA; rssharifi@gmail.com; 4 Biomaterials and Tissue Engineering Laboratory, Department of Chemical Engineering, University of South Carolina, Columbia, SC 29208, USA

**Keywords:** antiviral polymers, natural, synthetic, polysaccharides, nucleic acid polymers, dendrimers, sialylated polymers, viral infection

## Abstract

Polymers, due to their high molecular weight, tunable architecture, functionality, and buffering effect for endosomal escape, possess unique properties as a carrier or prophylactic agent in preventing pandemic outbreak of new viruses. Polymers are used as a carrier to reduce the minimum required dose, bioavailability, and therapeutic effectiveness of antiviral agents. Polymers are also used as multifunctional nanomaterials to, directly or indirectly, inhibit viral infections. Multifunctional polymers can interact directly with envelope glycoproteins on the viral surface to block fusion and entry of the virus in the host cell. Polymers can indirectly mobilize the immune system by activating macrophages and natural killer cells against the invading virus. This review covers natural and synthetic polymers that possess antiviral activity, their mechanism of action, and the effect of material properties like chemical composition, molecular weight, functional groups, and charge density on antiviral activity. Natural polymers like carrageenan, chitosan, fucoidan, and phosphorothioate oligonucleotides, and synthetic polymers like dendrimers and sialylated polymers are reviewed. This review discusses the steps in the viral replication cycle from binding to cell surface receptors to viral-cell fusion, replication, assembly, and release of the virus from the host cell that antiviral polymers interfere with to block viral infections.

## 1. Introduction

**Figure 1:**
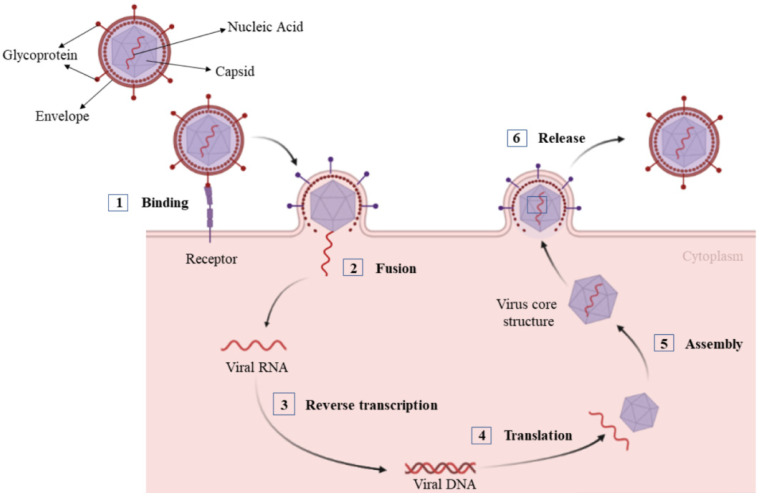
Steps in the viral replication cycle.

Due to their rapid mutation and evolution, viral infections have always been a global health challenge threatening both human and animal health. A virus is a nonliving microscopic structure that contains the genomic material for replication encapsulated and protected by a proteinaceous membrane. Unlike living organisms, viruses must invade a living cell, like a human or animal cell, and hijack its metabolic system for energy and nutrients to replicate and grow [1]. Some viruses even recruit the metabolic system of cancer cells to support their reproduction [2]. Some viruses are surprisingly simple like the Ebola virus, which is made up of only seven different proteins, even though this virus has had devastating consequences on human health [3,4]. Conversely, giant viruses like the Mimivirus possess genes for metabolizing proteins and serve as a bridge between nonliving viruses and living organisms [5]. Although there are considerable differences among virus types, viral replication consists of six basic steps as shown in Figure 1 [6]. These include (1) interaction between the proteins on the viral surface with surface receptors on the host cell for viral attachment; (2) fusion of the virus and host cell membranes for viral penetration in the host cell; (3) release of the viral genomic material inside the host cell by uncoating the virus; (4) synthesis of viral components like proteins, RNAs and DNAs by the host cell for viral replication; (5) assembly of the synthesized components into viral particles; and (6) exocytosis of the particles from the host cell to the interstitial space for viral spreading to other cells.

The function of an antiviral agent is to block one or more of the basic steps in the viral replication cycle without affecting the normal metabolic activities of the cell [7]. As viruses use the host cell for replication, it is challenging to develop safe and effective antiviral agents that do not affect the function of the host cell. As a result, many commercially available antiviral agents are limited by undesired side effects [8]. Further, the effectiveness of antiviral agents and vaccines is severely constrained by viral mutation [9,10].

Polymers, due to their tunable chemical structure and composition, high molecular weight, and their buffering effect possess unique capabilities as antiviral agents. Many polymers have been recently developed to meet the global demand for antiviral agents and to treat viral infections [11,12,13,14,15,16,17,18,19,20,21,22,23,24,25]. Although the mechanism of action of antiviral polymers is not completely understood, it is known that their potency and effectiveness can be tailored against a specific virus by varying the polymer molecular weight, chain architecture, composition, or functional groups [26,27]. Two approaches are used to utilize polymers as part of an antiviral system to fight against infections. In the first approach, the polymer is used as a matrix to protect, stabilize, and deliver the antiviral agent to the site of infection. In the second approach, the functionalized polymer is used as an antiviral agent to bind to the surface of viral particles to inhibit infections. The polymer functional groups that take part in binding to viral particles include among others phenolic, sulfate, amine, and carboxylic acid groups. The focus of this review is on the latter in which the polymer acts directly as an antiviral agent to fight against viral infections. Keeping an eye on current and future pandemics, this review covers repeating functional units in the structure of macromolecules that impart antiviral activity to natural and synthetic polymers.

## 2. Natural Polymers

**Figure 2:**
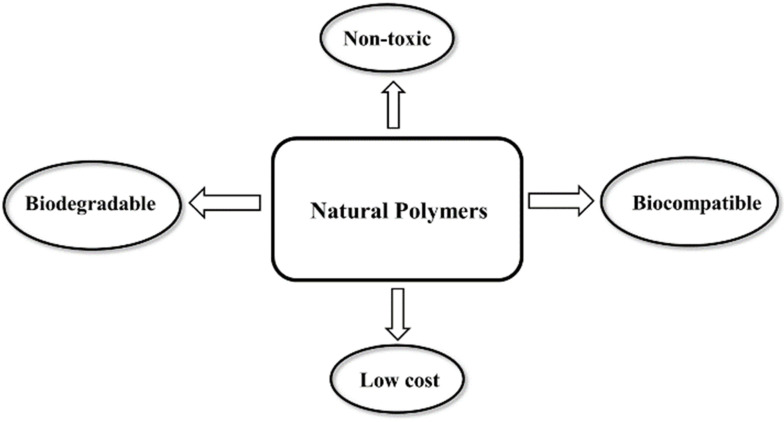
Key benefits of natural polymers as antiviral materials.

Natural polymers or biopolymers are classified into polysaccharides, polypeptides (proteins), and nucleic acid polymers (polynucleotides) [28]. Natural polymers as components of living systems are derived from plants, animals, and microorganisms [28]. Advantages of natural polymers over synthetic polymers include biocompatibility, non-toxicity, biodegradability, and intrinsic antiviral properties as shown in Figure 2 [29]. Among different classes of biopolymers, polysaccharides and nucleic acid polymers are capable of interfering with the virus-host cell interaction to block fusion, entry, and replication of the virus in the host cell [30].

### 2.1. Polysaccharides

Polysaccharides are an abundant source of renewable and biodegradable polymers. Polysaccharides are made up of more than 10 monosaccharide repeat units connected by glycosilic linkages in branched or linear chains with molecular weights ranging from tens of thousands to a few millions [31]. Like other natural polymers, polysaccharides play vital functions in living systems from cell adhesion to intracellular signaling and maintenance of biological and mechanical properties of tissues [32]. Natural plant polysaccharides are composed of long sequences of monosaccharides with different biological activities including anti-inflammatory, immunomodulatory, antioxidant, and antiviral activities [33,34]. Table 1 presents the general properties of carrageenan, chitin, chitosan, and fucoidan as widely studied polysaccharides with antiviral activities.

Polysaccharides possess different types of functional groups including hydroxyl, amine, and carboxylic acid groups which can be used for polymer modification [33,34]. These functional groups are used to tailor the properties of polysaccharides to specific biological applications [38,39]. The inhibitory effect of negatively charged polysaccharides on viral infections is well documented [40,41,42]. Polysaccharides interact with Trans-Activator of Transcription (Tat) regulatory protein of viruses to inhibit their interaction with cell surface receptors like heparan sulfates [43]. As an example, carrageenan which is a naturally sulfated polysaccharide of red algae, inhibits many viral infections including human papillomavirus (HPV), herpes simplex virus type-1 (HSV-1), and influenza A virus (IAV) by interacting with the viral Tat protein [44,45]. Numerous studies have reported the inhibitory effect of plant polysaccharides on replication of human and animal viruses including SARS associated coronaviruses (SARS-CoV) [46,47,48,49]. These include brown algae, *Auricularia auricula*, *Pinus massoniana*, and Acanthopanax polysaccharides [50]. Recently, the antiviral activity of highly sulfated synthetic oligomers and polymers has been investigated for inhibition of viral infections [23]. These studies show that both long and short chain, highly sulfated polysaccharides possess inhibitory effect on viral infections. According to these results, highly sulfated long-chain glycomimetic polymers prevented human papillomavirus (HPV16) infection in vitro and in vivo by interaction with the virus prior to cell attachment whereas highly sulfated short-chain glycomimetic oligomers inhibited viral infection post cell attachment [23]. The polyanionic nature of sulfated polysaccharides facilitates interaction with the positively charged domains of the virus envelope glycoproteins to form a non-reversible complex, thus making the viral glycoprotein sites inaccessible to interact with surface receptors of the host cell [51]. This non-reversible polysaccharide-viral interaction blocks binding and fusion of the virus with the host cell which inhibits viral entry in the cell [51]. Seaweed polysaccharides with high anionic sulfate content are attractive as antiviral agents to inhibit infections [52,53,54]. The structural diversity and complexity of marine polysaccharides and their derivatives contribute to their antiviral activity at different stages of viral infections [55]. The antiviral activity of polysaccharides is affected by their molecular weight. As the antiviral activity arises from the interaction of polysaccharides with viral envelope proteins through reversible secondary interactions like electrostatic, nonpolar, and hydrogen bonding, long chains that can form irreversible complexes substantially increase antiviral activity. In one study, polymeric sulfated carrageenan was more effective in blocking HPV16 viral infection in mice as compared to oligomeric sulfated carrageenan [23]. In another study, high molecular weight fucoidan polysaccharide showed antiviral activity against influenza A virus at low doses whereas low molecular weight fractions showed antiviral activity only at medium to high doses [56,57]. As a result, high molecular weight polysaccharides in general show higher antiviral activity [58].

#### 2.1.1. Carrageenan

**Figure 3:**
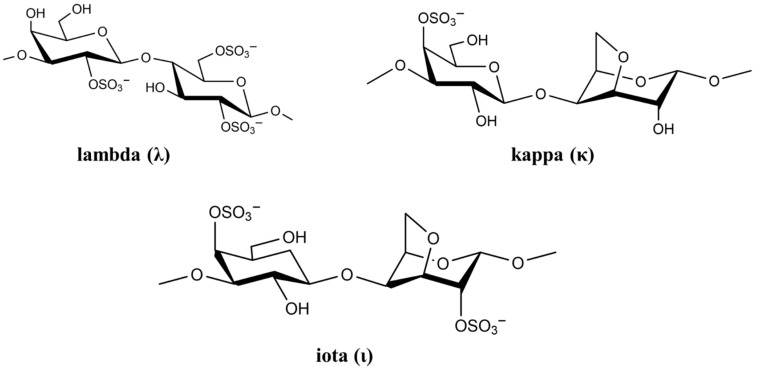
Chemical structure of lambda (λ), kappa (κ), and iota (ι) carrageenan.

The cell wall of marine red seaweed contains the polysaccharides agaran and carrageenan that form approximately 50% of the dry mass of seaweed. As a high molecular weight sulfated polysaccharide, carrageenan has long been used as a gelling agent and stabilizer in food [59]. However, carrageenan has many biological activities including anticoagulation, antioxidation, antiviral, and immunostimulatory [60]. Based on sulfate content, there are more than 10 types of carrageenan, but only the types lambda (λ), kappa (κ), and iota (ι) are of particular interest in viral and medical applications [61]. The chemical structure of the three carrageenan types is shown in Figure 3.

The negatively-charged sulfate groups of carrageenan neutralize the positive charge of the cell surface, leading to inhibition of viral attachment, penetration, and viral uncoating [62]. The biological activity of carrageenan strongly depends on its degree of sulfation, which depends on carrageenan (λ, κ and ι) as well as virus type [63]. List of studies on antiviral activity of λ, κ, and ι carrageenan are presented in Table 2.

In general, a higher degree of sulfation leads to a higher antiviral activity [55]. It is reported that λ-carrageenan having 35% sulfation exhibits approximately ten times higher inhibition against HIV infection as compared to κ-carrageenan with 25% sulfation [69]. Aside from sulfation, the source of polysaccharide affects its antiviral activity [70]. Carrageenan is capable of affecting virus replication directly through the sulfate groups leading to a decrease in viral activity [71]. For example, carrageenan binding changes the structure of HSV virus, namely the structure of glycoproteins B and C, which results in virus deactivation. Vissani et al. demonstrated that the antiviral activity of ι-carrageenan against herpesvirus-3 is linked to envelope glycoprotein binding [72]. Functional characterization showed that ι-carrageenan and *N*-sulfonated derivatives of poly (allylamine hydrochloride) blocked the release of human metapneumovirus (hMPV) from infected cells, which reduced viral spreading [73]. A nasal spray for the delivery of carrageenan has been developed for treating patients infected with rhinovirus (HRV), coronavirus (HCoV), and influenza A (IAV) virus. The nasal spray decreased viral symptoms in infected patients and it was most effective against the human coronavirus [74]. It should be pointed out that ι-carrageenan is an approved antiviral drug for use against not only HRV, IAV-H1N1 and HCoV-OC43 but also against viral throat infection [75]. A study on the use of ι-carrageenan against viral throat infection showed 85% and 91% decrease in symptoms in IAV and HCoV-OC43 infected patients, respectively [75].

**Figure 4:**
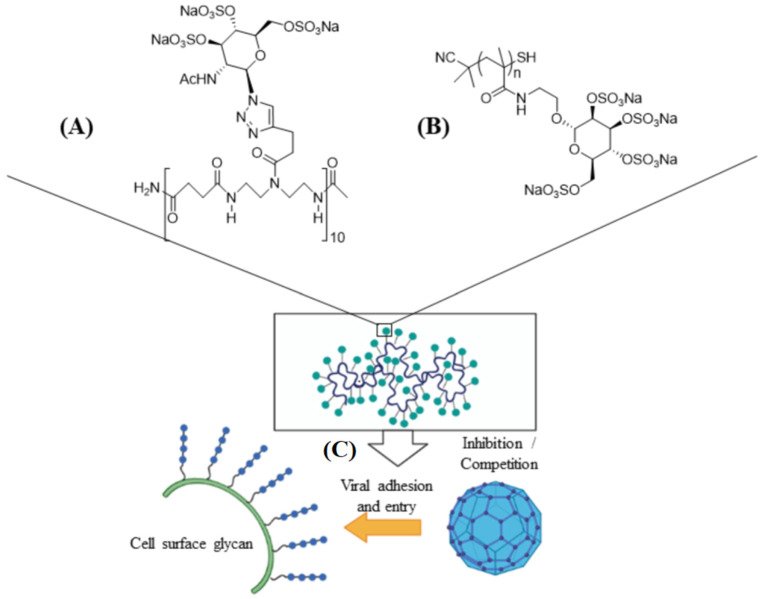
Schematic representation of short ((**A**), 10 sugar units) and long ((**B**), n units) chain oligomeric sulfated saccharides to mimic different properties of glycopolymers; (**C**) the sulfated glycopolymers block viral infection by interfering with virus attachment to the cell surface. Reprinted with permission from Ref. [23]. Copyright 2020, American Chemical Society.

Carrageenan has also been used against human papillomavirus (HPV) which is a non-enveloped small DNA virus capable of infecting mucosal membrane or skin [76]. In one study, carrageenan showed antiviral activity against HPV pseudovirus in a luciferase mouse model without exhibiting a cytotoxic effect [77]. In another study, the antiviral activity of carrageenan was compared with heparin sulfate and highly sulfated synthetic glycomimetic polymers in vitro and in vivo [23]. Both short chain oligomeric sulfated saccharides with 2–10 sugar units and long polymeric saccharides with 40–80 sugar units were synthesized to mimic different properties of glycopolymers, as shown in Figure 4A,B. The in vitro evaluation of the antiviral agents were done with HPV16-eGFP pseudovirus incubated with HeLa cervical cancer cells whereas the in vivo evaluations were done with HPV16-luciferase inoculated in the mouse vagina [23]. The addition of carrageenan, heparin sulfate, and polymeric sulfated saccharides inhibited the attachment of HPV16 pseudovirus to the surface of HeLa cells to block viral infection (Figure 4C) [23]. The effectiveness of polymeric sulfated saccharides with 40 units in inhibiting viral attachment was slightly less than that with 80 units. The in vivo imaging results showed that both carrageenan and heparin sulfate were effective in inhibiting HPV16 infection after 2 days of inoculation [23]. According to in vivo imaging results, the polymeric sulfated glycomimetic saccharides were effective in blocking the activity of HPV16 virus whereas the oligomeric saccharides were ineffective [23]. Carrageenan was effective in reducing viral infection to below 50% at concentrations as low as 100 ng/mL [23]. The results demonstrate the importance of molecular weight of sulfated polysaccharides in blocking viral infections.

Recently, the antiviral activity of different carrageenan types against SARS-CoV-2 has been reported [68,78,79]. In one study, it was shown that λ-carrageenan purified from red seaweed was effective against influenza A and B and SARS-CoV-2 [78]. In another study, a carrageenan nasal spray inhibited SARS-CoV-2 infection in Vero E6 monkey kidney epithelial cells expressing TMPRSS2 receptor, which was attributed to the polyanionic nature of ι- and κ-carrageenan [79]. In another study, ι-carrageenan delivered using a nasal spray inhibited the entry of SARS-CoV-2 spike protein pseudo-typed lentiviral particles to Vero B4 embryonic monkey kidney cells [68]. In that study, ι-carrageenan also showed effectiveness against rhinoviruses and endemic coronaviruses with an IC_50_ value of 2.6 µg/mL [68]. These studies show that ι-carrageenan can potentially serve as a prophylactic agent in the treatment of pandemic outbreaks of new viruses in the absence of vaccines.

#### 2.1.2. Chitosan and Its Derivatives

**Figure 5:**
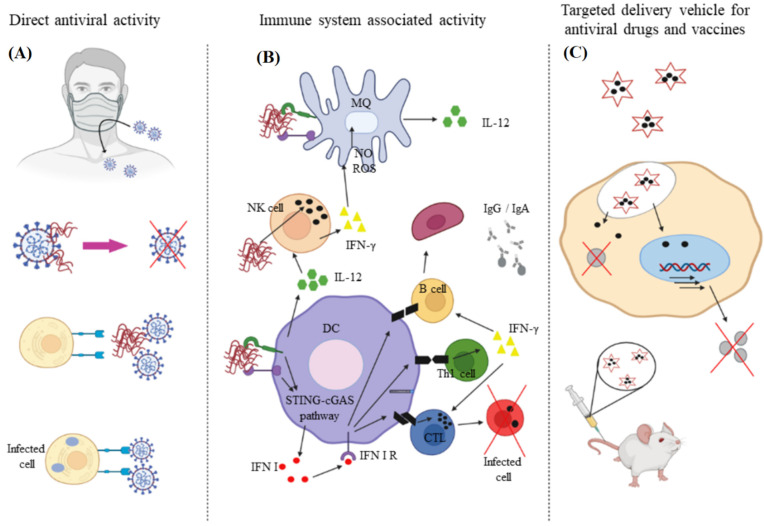
The direct (**A**) and indirect (**B**) approaches to using chitosan to inhibit viral infection; (**C**) chitosan is also used as a carrier for delivery of antiviral agents. Reprinted with permission from Ref. [83]. Copyright 2021, Elsevier.

Chitin and chitosan due to their biocompatibility, degradability, and non-immunogenicity are used in the development of antimicrobial wound dressing as well as other medical applications [80,81]. Chitin, comprised of *N*-acetyl-*D*-glucosamine and *D*-glucosamine units, is isolated from the shell of arthropods like shrimp, lobster, crab, and insects or produced by fermentation [82]. Chitosan is produced from chitin by partial deacetylation or by enzymatic hydrolysis [83]. Although the antimicrobial properties of chitosan is well-documented, less is known about the antiviral activity of this biopolymer [84]. The degree of acetylation and amination, chitosan concentration, molecular weight, overall charge as well as charge distribution, and functional group modification affects antiviral activity of chitosan [85,86]. The positively charged derivatives of chitosan possess antiviral activity whereas the negatively charged derivatives are not antiviral [81]. Chitosan can inhibit viral infection directly and indirectly or it can be used as a carrier in the delivery of antiviral agents, as shown in Figure 5 [87]. In the direct approach, chitosan interferes with the receptors that maintain viral activity and growth [87]. For example, the anionic sulfate groups on glucosamine residues of chitosan interact electrostatically with positively-charged envelope glycoprotein GP120 on the virus surface to prevent its fusion with the host cell, which effectively inhibits virus entry and replication [88]. In the indirect approach, chitosan mobilizes the immune response by stimulating proliferation and activation of macrophages and natural killer cells against the invading virus. In this regard, the *N*-acetyl-glucosamine residues in chitosan increase the level of reactive oxygen species in macrophages after uptake, which subsequently increase the synthesis of IFN-γ by lymphocytes in the spleen, which in turn prevent translation of viral genomic RNA [89].

**Figure 6:**
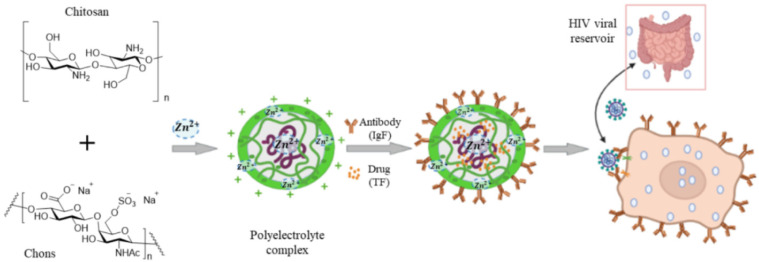
A nanocomplex for delivery of tenofovir based on zinc-stabilized chitosan and chondroitin sulfate. Abbreviations are TF for tenofovir, CS for chitosan, Chons for chondroitin sulfate, IgA for antibody, PECs for polyelectrolyte complexes, and PBMCs for human peripheral blood mononuclear cells. Reprinted with permission from Ref. [95]. Copyright 2016, American Chemical Society.

A sulfated derivative of chitin, namely *N*-carboxymethyl chitosan-*N*-*O*-sulfate (NCMCS), was shown to inhibit uptake and replication of human immunodeficiency virus-1 (HIV-1) in CD4^+^ human T-lymphoma cells, which was attributed to the interference of NCMCS with the interaction of viral GP120 envelope glycoproteins with CD4^+^ cell surface receptors [90]. In another study, a sulfated derivative of carboxymethyl chitin (SCM-chitin) was shown to inhibit penetration and subsequent pathogenesis of herpes simplex type-1 virus (HSV-1) in vitro in monkey kidney epithelial cells (Vero) as compared to carboxymethyl chitin which showed no activity [91]. The sulfated derivative of chitosan was also found to protect Vero cells from cytopathic infection by Coxsackie and Rift Valley fever RNA viruses [92]. Chitosan and its derivatives are used as a carrier in delivery of antiviral drugs to reduce the minimum required dose and increase bioavailability, thus improving the drugs’ therapeutic effectiveness (Figure 5C) [93]. In one study, curcumin encapsulated in chitosan nanoparticles (NPs) was shown to have higher antiviral activity against hepatitis C virus genotype 4a (HCV-4a) in human hepatoma cells as compared to unencapsulated curcumin [94]. A delivery system based on zinc-stabilized nanocomplex of chitosan and chondroitin sulfate for delivery of tenofovir was developed for inhibition of HIV-1 viral infection, as shown in Figure 6 [95]. The tenofovir-loaded nanocomplex showed strong antiviral activity against HIV-1 in human peripheral blood mononuclear cells (PBMCs) without a cytotoxic effect. The delivery system showed considerable antiviral activity in the absence of tenofovir [95].

**Figure 7:**
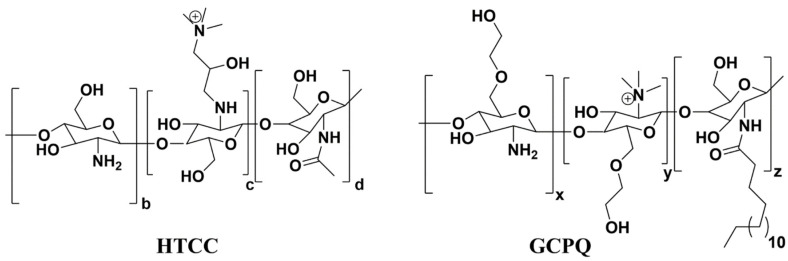
Chemical structures of *N*-(2-hydroxypropyl)-3-trimethylammonium chitosan (HTCC) and *N*-palmitoyl-*N*-monomethyl-*N*,*N*-dimethyl-*N*,*N*,*N*-trimethyl-6-*O*-glycolchitosan (GCPQ) with quaternary ammonium functional groups.

Recently, the antiviral activity of quaternary ammonium modified chitosan such as *N*-(2-hydroxypropyl)-3-trimethylammonium chitosan (HTCC) and *N*-palmitoyl-*N*-monomethyl-*N*,*N*-dimethyl-*N*,*N*,*N*-trimethyl-6-*O*-glycolchitosan (GCPQ) is reported against SARS-CoV-2, as shown in Figure 7 [96,97]. Due to the quaternary ammonium functional groups in the structure of HTCC and GCPQ, these positively charged molecules bind electrostatically to coronavirus S proteins to block not only viral entry to host cells but also to prevent viral replication. List of studies on antiviral activity of chitosan and its derivatives are presented in Table 3.

#### 2.1.3. Fucoidan

**Figure 8:**
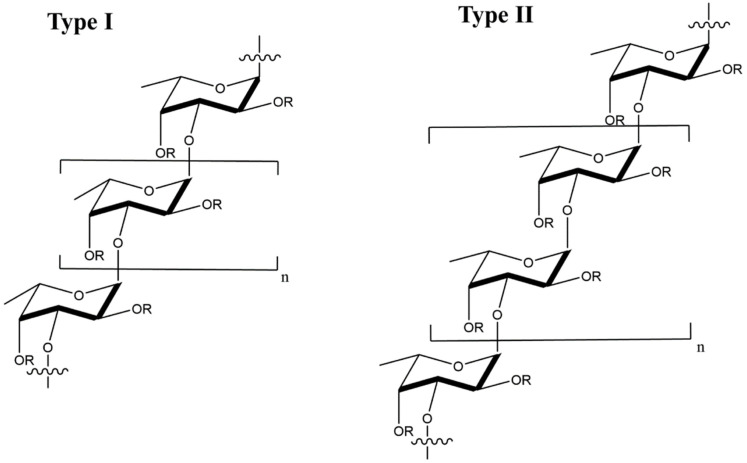
Molecular structure of type I and type II fucoidans from brown seaweed; the R groups represent sulfate, glucuronic acid, or fucopyranose groups.

Fucoidan is a sulfated, acidic polysaccharide composed of L-fucose and galactose monosaccharides with smaller proportions of xylose and mannose [102]. It is isolated from the cell wall of marine brown seaweeds like *Ascopbyllum nodosum*, *Fucus vesiculosus*, *Saccharina japonica*, and *Sargassum thunbergia*. Based on molecular structure, there are two types of fucoidan, as shown in Figure 8, namely type I having (1→3)-l-fucopyranose repeats and type II having (1→3)- and (1→4)-l-fucopyranose repeat units [103]. Depending on degree and pattern of sulfation, molecular weight and composition, fucoidans possess different biological properties including antiviral [56,57,104,105,106,107], anti-inflammatory [108], immunologic [109] and antitumor [110,111] properties.

The mechanism of action for antiviral activity of fucoidans from different sources has been recently studied [56,112]. Fucoidan inactivates the virus before or after cell internalization [113]. After internalization, fucoidan prevents viral transcription as well as other intracellular targets, thus blocking viral replication [113]. List of studies on antiviral activity of fucoidans from different sources are presented in Table 4. In one recent study, *Kjellmaniella crassifolia* fucoidan with a molecular weight of 540 kDa and 30% sulfate content was targeted to inhibition influenza A virus (IAV) [56]. According to in vitro results, the fucoidan outperformed the anti-IAV drug amantadine in inhibiting viral infection [56]. In another study, low molecular weight (LMW) fucoidans with 30–42% fucose, 19–24% galactose, 3–5% uronic acid, and 30–32% sulfate were synthesized and their antiviral activity was evaluated [57]. The LMW fucoidans showed antiviral activity in HeLa, Hep-2, and MDCK cells infected with influenza A virus at medium and high fucoidan doses in the range of 0.15–2.4 mg/mL whereas low doses were ineffective against influenza A [57]. Further, the survival time of the virus-infected mice increased and their lung index improved with intravenous administration of fucoidan [57]. In another study, the influence of *Fucus evanescens* fucoidan on inhibition of hepatitis B virus (HBV) was investigated in an HBV-infected mouse model [114]. In this study, fucoidan acted as an adjuvant to suppress HBV DNA, HBV surface antigen (HBsAG) and active HBV proteins implicated in viral replication, namely HBeAg and HBcAg [114]. The treatment of HBV-infected mice with 100 mg of fucoidan upregulated the expression of interferon-α (INF-α) via activation of mitogen-activated protein kinase (MAPK-ERK1/2) pathway, which reduced DNA production and viral replication [114]. Despite numerous studies demonstrating the therapeutic potential of fucoidan for treating HBV infections, further research is needed to validate the results in large animal models prior to translation to clinical practice.

### 2.2. Nucleic Acid Polymers

**Figure 9:**
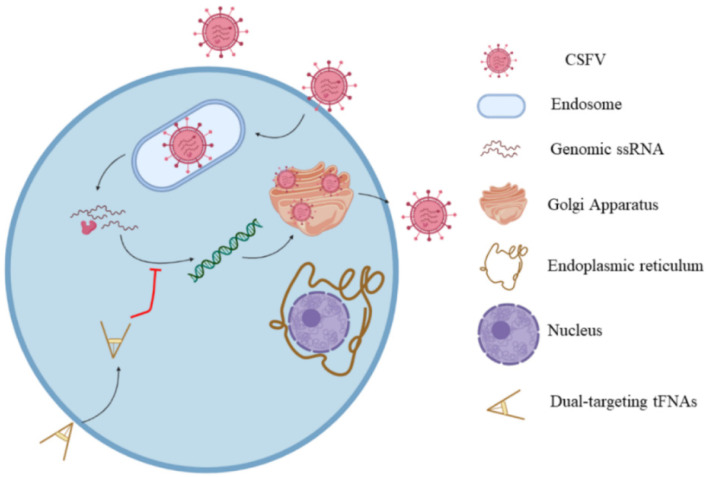
Schematic illustration of the mechanism of siRNA delivery by tFNA nanomaterial. Reprinted with permission from Ref. [134]. Copyright 2021, American Chemical Society.

Nucleic acid polymers (NAPs), as phosphorothioate oligonucleotides, possess an-tiviral activity [118]. Oligonucleotides and NAPs can be stabilized against degradation by nucleases made amphipathic by phosphorothioation of non-bridging oxygen atoms through phosphodiester linkages [118]. The antiviral activity of phosphorothioated NAPs was first reported in a study on mechanism of action and the effect of NAPs’ structure on inhibition of HIV-1 infection [114]. It was found that NAPs inhibited viral entry to the host cell and the extent of inhibition was controlled by the size of NAPs, not their sequence distribution. According to this study, NAPs with >20 nucleotides showed antiviral activity [114]. The antiviral activity of NAPs against HIV-1 was at-tributed to the interaction of NAPs with the amphipathic alpha helix triplets found in the core of metastable envelope glycoprotein gp41 of HIV to inhibit viral entry to the host cell. The first step in the viral inhibition is decomplexation of the amphipathic al-pha helices of gp41 by NAPs followed by interaction with hydrophobic moieties of the alpha helices, which explains the dependence of NAP inhibition on size and phos-phorothioation [119]. The interaction of NAPs with amphipathic alpha helix triplets of gp41 was used for targeting and inactivating other viruses including cytomegalovirus, herpesvirus, and lymphocytic choriomeningitis virus [120,121,122,123]. A similar mechanism is proposed for the role of NAPs in blocking the entry of hepatitis C virus (HCV) into host cells [122]. However, one study reported that NAPs did not prevent HCV attach-ment to the host cells but blocked the post-binding step required for viral entry [123]. The post-binding step is posited to involve the interaction of NAPs with other viral proteins including the hypervariable region of glycoprotein E2 or apolipoprotein E [124,125]. The antiviral activity of NAPs assessed against ductal hepatitis B virus (DHBV) with primary duck hepatocytes (PDH) as the host was shown to be dependent on NAP size and amphipathicity [126]. Specifically, NAPs with >40 nucleotides showed higher antiviral activity against DHBV. The therapeutic potential of two NAPs, namely REP 2055 and REP 2139-Ca, were evaluated in patients with HBeAg positive chronic HBV infection in two clinical studies [127,128]. The patients treated with NAP monotherapy showed 2–7 log reduction in serum HBsAg, 3–9 log reduction in serum HBV DNA, and the presence of serum anti-HBsAg antibodies in the patients’ blood [127,128]. These results indicated that NAPs could potentially be used as a component in combination therapies in treating patients with HBV. In another study, the safety and therapeutic efficacy of REP 2139 and REP 2165 NAPs combined with commonly used medication to treat patients with chronic hepatitis B, namely tenofovir disoproxil fumarate (TDF) and pegylated interferon α-2a (PegIFN), were assessed in a randomized phase 2 clinical trial. The REP 2139 NAP and its bioequivalent REP 2165 block the assembly of subviral particles (SVPs) in hepatocytes resulting in clearance of HBsAg surface antigens. The experimental NAP groups were TDF+PegIFN+REP 2139 and TDF+PegIFN+REP 2165 [129]. According to the results, there was no difference in the levels of HBsAg, indicative of HBV infection, anti-HBs, indicative of recovery from HBV infection, and HBV DNA between the two NAP groups [129]. The undesired side effects induced by PegIFN, namely thrombocytopenia and neutropenia, were unaf-fected by the addition of NAPs to TD and PegIFN. However, the levels of HBV-induced transaminases were initially higher in the NAP groups, but the levels returned to nor-mal during therapy and follow-up [129]. Treatment with TDF+PegIFN without NAP resulted in low HBsAg loss and HBsAg seroconversion as well as low functional cure. Conversely, treatment with TDF+PegIFN+NAP resulted in high HBsAg loss and HBsAg seroconversion as well as high functional cure with normal liver function [129]. No differences in HBsAg loss, seroconversion, or functional cure were observed between the two NAP groups. An in vitro model based on HepG2.2.15 hepatocyte cells that constitutively express HBV demonstrated that the NAPs’ endosomal release selectively impaired the secretion of HBsAg without accumulation of HBsAg in the cells [130]. NAPs due to their biocompatibility, degradability, and their ability to penetrate cell membranes have been used as a carrier for delivery of antiviral drugs against classical swine fever virus (CSFV) in infected pigs [131,132]. The enveloped virus with a sin-gle-stranded RNA causing CSFV belongs to the genus Pestivirus in the family Fla-viviridae [133]. The small interfering RNA (siRNA) was delivered to the host cells in a porcine CSFV model using tetrahedral framework nucleic acid (tFNA), as a three-dimensional DNA nanomaterial that self-assembles by complementary base pairing [134]. Concurrent delivery of C3 and C6 siRNAs in self-assembled tFNA to host cells prevented viral replication and exocytosis, as shown in Figure 9 [134].

Following penetration through the cell membrane, CSFV is transported to the endoplasmic reticulum where its single-stranded RNA is released for protein translation. Following RNA translation, CSFV is replicated on the cytoplasmic membrane leading to the formation of viral particles in the Golgi apparatus. The siRNAs carried by tFNA degrade the released viral RNA to prevent its translation in the endoplasmic reticulum. List of studies on antiviral activity of NAPs are presented in Table 5.

## 3. Synthetic Polymers

Polymers are chain-like molecules formed by polymerization of small, reactive organic molecules. Polymers due to their long chain length or high molecular weight are used extensively in surface coating applications because each polymer chain can make many physical bonds to irreversibly coat the surface [29,135]. As a result, polymers are very attractive as an antiviral material to bond irreversibly to viral glycoproteins to cover and conceal the viral surface, thus blocking the interaction of viral particles with the host cell [11,12,13,14,15,16,17,18,19,20,21,22,23,24,25]. Unlike natural polymers, the chemical composition, functional group type and extent of functionalization, molecular weight, charge density and distribution, degradation and stability of synthetic polymers can be engineered to maximize antiviral activity against a specific virus type [22,136,137]. Among synthetic polymers, dendrimers and sialyl-based polymers have been extensively studied as antiviral agents to fight against infections.

### 3.1. Dendrimers

**Figure 10:**
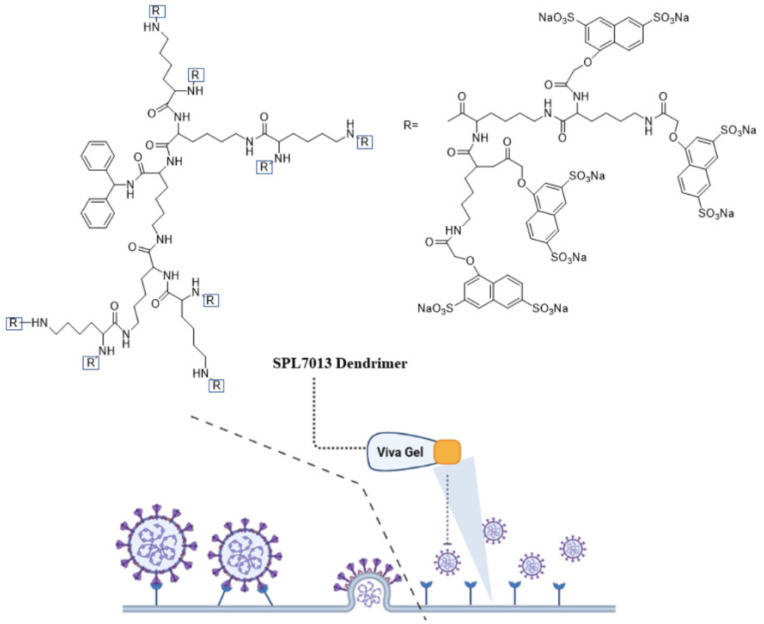
Chemical structure and mechanism of action of VivaGel^®^ as an antiviral agent. The image was created by BioRender software.

Dendrimers due to their nanoscale size and structural uniformity are attractive as a carrier for delivery of antiviral agents [138]. The branched structure of dendrimers results in spherical and symmetric nanostructures with a monodisperse size in the 2–10 nm range [139]. The dendrimer generation which is the number of branches or the number of repeated synthetic cycles, controls the size of dendrimers [139]. The chemical groups on the dendrimer surface can be functionalized to produce polycationic or polyanionic dendrimers [139,140]. Peptides and drug molecules can be conjugated to dendrimers via cleavable linkages to form functional dendrimers with controlled biological activity [141]. Antiviral activity of dendrimers is attributed to their multivalency, symmetrical and monodisperse molecular structure, and the presence of multiple functional groups on the dendrimer surface. The dendrimer-based antiviral agents act mechanistically by interfering with the interaction of virus with the host cell [142]. As a result, antiviral activity depends on dendrimer molecular weight and type of functional groups present on the dendrimer surface [142]. Among different types, peptide-conjugated dendrimers show excellent antiviral activity against many virus types. A sulfonated derivative of polylysine dendrimer with a benzhydrylamine core was used to prevent infection against intravaginal herpes simplex virus (HSV) and simian immunodeficiency virus/human immunodeficiency virus (SIV/HIV) chimeric viruses [143,144]. In vitro studies with monkey kidney epithelial cells (Vero) demonstrated that this dendrimer completely prevented adsorption and penetration of the virus in host cells and inhibited DNA synthesis in the infected cells [144]. Figure 10 shows the chemical structure and mechanism of action of an FDA approved VivaGel^®^ dendrimer (SPL7013), which is used in preventing genital herpes (HSV-2) and HIV. The VivaGel^®^ is composed of anionic G4-poly(L-lysine) dendrimer with a benzylhydramine-amide-lysine core with 32 sodium (1-naphthyleneyl-3,6-disulphonic acid)-oxyacetamide functional groups [145]. The functional groups on the dendrimer surface act to prevent infection by blocking the virus from binding to host cells [145].

Paull and coworkers evaluated the antiviral activity of SPL7013 dendrimer against SARS-CoV-2 [146]. Compared to polyanionic biopolymers such as io-ta-carrageenan and heparin (as linear sulphated molecules), the branched sulfonated structure of SPL7013 dendrimer inhibited SARS-CoV-2 infection in Vero E6 cells early in the replication cycle by blocking viral entry to the cells. In another study, a new class of dendrimers with tryptophan (Trp) functional groups on the surface were synthe-sized and their antiviral activity was evaluated against HIV and enterovirus-71 (EV71) [147]. Dendrimers with different surface amino acid groups including aromatic and non-aromatic, decarboxylated Trp (tryptamine), and methylated Trp were synthe-sized to study the relation between amino acid structure and antiviral activity [147]. According to the results, Trp-conjugated dendrimers act mechanistically by interacting with cell surface glycoproteins to interfere with virus-cell interaction early in the HIV replication cycle prior to viral entry in the host cell [147]. The type of peptide and its structure (aliphatic versus aromatic) affected antiviral activity of the dendrimer. Dendrimers conjugated with aromatic amino acids like tryptophan showed antiviral activity against both HIV and EV71 whereas dendrimers conjugated with aliphatic amino acids like alanine were inactive. The natural peptide LL-37 which is a member of cathelicidin family of peptides show antiviral activity against the respiratory syn-cytial virus (RSV), which is a common lower respiratory tract pathogen in children [148]. However, LL-37 peptide has limited stability in physiological medium. In a re-cent study, linear (SA-35) and hyperbranched (dendrimeric, LTP) cationic peptides from the helical region of LL-37 peptide were synthesized and their antiviral activity were compared with the natural peptide [149]. The results showed that the linear and hyperbranched peptides had higher antiviral activity as compared to the natural pep-tide which was attributed to destabilization of the viral envelope and competitive in-hibition of virus attachment/fusion with the host cell.

A range of polycationic and polyanionic dendrimers have been synthesized and evaluated against viral infections [150]. The antiviral activity of these dendrimers is due to electrostatic interaction between the viral envelope proteins and the den-drimer’s ionic functional groups [151]. In one study, polycationic dendrimers with *N*-alkylated 4,4′-bipyridinium units of varying charge density were synthesized and evaluated with respect to inhibition of HIV-1 [152]. Results showed that the inhibition of HIV-1 in MT-4 host T cells by the dendrimers was mediated by CXCR4 coreceptor and was independent of charge density of dendrimers. In another study, a polyanionic carbosilane dendrimer (PCD), designed to inhibit HIV-1 infection, was used to prevent viral infections causing sexually transmitted diseases [153]. The PCD dendrimer inhib-ited herpes simplex virus-2 (HSV-2) infection by reducing plaque formation in HSV-2 infected Vero cells, which was attributed to direct binding between the sulfonate groups on the dendrimer surface and glycoprotein B of the virus [154]. In another study, a PCD consisting of a polyphenolic core with 24 sulfonate groups on the den-drimer surface was shown to possess antiviral activity against hepatitis C virus (HCV) at low and high concentrations [154]. In another study, PCDs with surface sulfonate or naphthalene sulfonate functional groups prevented not only HIV-1 infection by inter-fering with cell-virus fusion but also inhibited cell-to-cell transmission [138]. List of studies on antiviral activity of dendrimers against different viruses are presented in Table 6.

### 3.2. Sialyl-Based Polymers

**Figure 11:**
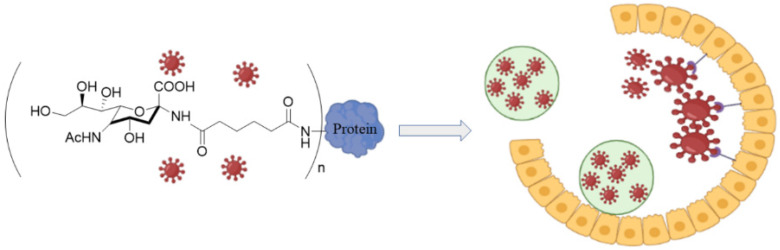
Schematic representation for the clearance of influenza virus by the mucin layer or mucin-mimetic glycol-conjugated proteins prior to uptake by epithelial cells. The image was created by BioRender.com software.

The sialylated glycans in the protective mucin layer of the nasal cavity disrupts the entry of influenza virus (IFV) particles to host cells by complexation with viral hemagglutinin (HA) [156]. The mucin barrier is the first line of defense in the immune system that traps and clears the viral particles upon mucin turnover, as shown in Figure 11 [157]. Although sialic acid (SA) and derivatives of SA have a strong affinity for the viral HA, the monovalent interaction between HA and SA is relatively weak [158]. Therefore, polymers modified with SA that can overcome the low interaction energy by complexation with multiple SA groups can effectively inhibit IFV infections [159]. In one study, the antiviral activity of a multivalent polymer conjugate consisting of poly (methyl vinyl ether-alt-maleic anhydride) modified with thiosialoside against PR8 influenza strain was comparable to those of Zanamivir^®^ and Oseltamivir^®^ drugs, as measured by growth inhibition assay [160]. The antiviral activity was attributed to multivalent binding of the modified polymer to neuraminidase on the viral particles to inhibit enzymatic activity, leading to viral particle aggregation and inhibition of virus-cell fusion. These results imply that polymers functionalized with monomeric sialoside groups which possess neuraminidase inhibitory activity can serve as an antiviral scaffold against early and late stages of influenza infection.

Glycopolymers based on sialyl oligosaccharides with a range of chain lengths and sugar densities were synthesized by reversible addition-fragmentation chain transfer polymerization (RAFT) for interaction with hemagglutinin on the surface of influenza virus [161]. The extent of interaction and molecular specificity of the copolymers against different types of influenza virus depended on sugar units (6′- versus 3′-sialyllactose), sugar density, and polymer chain length [161]. In another study, tri-arm star glycopolymers with homogenous number of arms consisting of inert spacers and glycomonomer segments were synthesized by a core-first approach. The star glycopolymers had three arms with each arm having a degree of polymerization ranging from 30 to 100 to match the sugar-binding pockets of hemagglutinin on influenza virus [162]. The inert core segment was based on poly(N,N-dimethylacrylamide) (PDMA) whereas the arms were made of 6′-Sialyllactose glycomonomer which served as a natural ligand for HA binding in human influenza virus [162]. The use of arms with different degrees of polymerization allowed tunning the ligand-receptor interaction to the desired strength for surface HA in influenza virus. In another study, a polymer brush consisting of side chains ending in α-2,6-linked sialic acid (SA) was synthesized by protection-group-free, ring-opening metathesis polymerization (ROMP) to enhance multivalent interaction as determined by hemagglutination binding [163]. In this work, the anomeric hydroxyl group of 6′-sialyllatose in the oligosaccharide was activated and converted to an azide by nucleophilic substitution to form an azido oligosaccharide. Next, the azido oligosaccharide was reacted with α-norbornenyl, ω-acetylene terminated PEG, prepared from amine-terminated hydroxyl PEG, using a copper-catalyzed click reaction. Then, the α-norbornenyl terminated oligosaccharide PEG was polymerized by ROMP to produce a brush polymer with oligosaccharide side chains terminated with SA [163]. According to the results, brush polymers with high degree of polymerization, high SA density, and long side chains produced strongest antiviral activity as measured by hemagglutination inhibition assay [163]. These brush polymers are useful as a model system to study the antiviral properties of native mucins with the passage of influenza A virus (IAV) through the epithelial mucin barrier as measured by the strength of interaction between the viral HA and the sialyloligosaccharides of mucin (Figure 11) [164]. List of studies on antiviral activity of sialylated polymers are presented in Table 7.

## 4. Conclusions

Polymers provide enormous opportunity to tailor antiviral activity via specific interaction with glycoproteins on the viral surface by varying molecular weight, type and degree of functionality, sequence distribution, extent of branching, and molecular architecture. Polymers can be used to increase bioavailability and therapeutic effectiveness of antiviral agents and reduce their undesired side effects. Natural biopolymers with multiple functional groups like carrageenan, chitosan, and fucoidan are used directly to bind and form irreversible complexes with envelope glycoproteins on the viral surface to block virus-host cell interaction, fusion, and entry to the host cell. Biopolymers are also used to mobilize the immune response against the invading virus by stimulating the activation of macrophages and natural killer cells. Natural nucleic acid polymers are used as antiviral agents after phosphorothioation to render them amphipathic and stabilization against nuclease degradation. Polymers deactivate the virus before cell internalization by inhibiting virus-host cell binding or after cell internalization by blocking virus replication. Synthetic dendrimers due to their symmetry, monodisperse molecular structure, and multifunctionality bind with high affinity to envelope glycoproteins on the viral surface to block virus-host cell interaction and fusion. Brush polymers modified with sialic acid groups bind with high affinity to viral hemagglutinin to form complexes that block viral infection. Research results have demonstrated that natural and synthetic polymers are useful as broad-spectrum antiviral agents to fight against many different types of viral infections. Further preclinical research in animal models and clinical trials are needed to validate the antiviral activity of polymers to fight new viral infections in the absence of vaccines.

## Figures and Tables

**Table 1:** Polysaccharides, their origin, and molecular characteristics.

Polysaccharide	Origin	Occurrence/Function	Molecular Characteristics	Ref.
Carrageenan	Algae	Structural polysaccharides of marine red algae	Heteropolysaccharide, linear, anionic	[35]
Chitin/chitosan	Animal	Chitin: structural polysaccharide from exoskeleton of insects and shells of crustaceans Chitosan: derivative of chitin prepared by deacetylation	Chitin: homopolysaccharide, linear, neutral Chitosan: heteropolysaccharide, linear, cationic	[36]
Fucoidan	Algae	Structure and composition dependent on species with diverse structures	Heteropolysaccharide, anionic, linear.	[37]

**Table 2:** Antiviral activity of λ, κ, and ι carrageenan against different viruses.

Type	Virus	Genome	Cell Line	IC50/EC50 (µg/mL)	Ref.
κ	DENV-1	RNA	Vero	>50 (EC50)	[64]
κ	DENV-2	RNA	Vero	1.8 (EC50)	[64]
κ	HSV-1	DNA	Vero	1.9 (EC50)	[65]
κ	HSV-2	DNA	Vero	1.6 (EC50)	[65]
κ	HIV-1	RNA	MT-4	12 (IC50)	[66]
ι	DENV-1	RNA	Vero	41 (EC50)	[64]
ι	DENV-2	RNA	Vero	0.40 (EC50)	[64]
ι	H1N1	RNA	MDCK	0.39 (IC50)	[67]
ι	SARS-CoV-2	RNA	Vero	0.05 (IC50)	[68]
λ	HIV-1	RNA	MT-4	1.9 (IC50)	[66]
λ	H1N1	RNA	MDCK	0.04 (IC50)	[44]
λ	VSV	RNA	HeLa	4.0 (IC50)	[66]

**Table 3:** Antiviral activity of chitosan and its derivatives.

Chitosan	Virus	Cell Line	Concentration	Ref.
Zinc stabilized chitosan	HIV-1	Human peripheral blood mononuclear	0.1–0.2% *w*/*v* chitosan in acetic acid	[95]
Curcumin-loaded chitosan nanocomposite	Hepatitis C	human hepatoma	20 µg/mL curcumin in 4% chitosan composite	[94]
Chitosan with medium molecular weight	AD169 strain of HCMV	Human embryonic lung fibroblasts	9–14 mg Fostcanet antiviral drug in 4.5 mg chitosan	[98]
Silver-loaded chitosan	H1N1 Influenza A	Madin-Darby canine kidney	Ag NPs in 10 mg/mL chitosan	[99]
Nanosilver-loaded polyquaternary phosphonium oligochitosan	Hepatitis A	Vero	100 µL/mL silver NPs	[100]
3,6-O-sulfated chitosan	HPV	293FT, Hela, HaCaT	2.4, 4.7, 4.2 µg/mL chitosan	[101]
HTCC	SARS-CoV2	human airway epithelium (HAE)	12.5 µg/mL HTCC	[96]
GCPQ	SARS-CoV2	Vero E6 and A549	10 µg/mL GCPQ	[97]

**Table 4:** Antiviral activity of fucoidans from different species of seaweed.

Fucoidan	Virus	Cell Line	IC50 (μg/mL)	Ref.
*Cladosiphon okamuranus*	Newcastle Disease (NDV)	Vero	0.75	[115]
*Scytosiphon lomentaria*	HSV-1, HSV-2	Vero	1.12–1.22	[112]
*Sargassum henslowianum*	HSV-1, HSV-2	Vero	0.48–0.89	[113]
Sporophyll of *Undaria pinnatifida* (Mekabu)	Influenza A	MDCK	15	[116]
Brown Algae: *Fucus evanescens*	HSV-1, HSV-2, HIV-1	MT-4	25–80	[117]

**Table 5:** Antiviral activity of NAPs against different viruses with their mechanism of actions.

NAP	Virus	Cell Line	Mechanism of Action	Ref.
Phosphorothioate Oligonucleotides	HIV-1	H9	Inhibiting viral entry to the host	[119]
amphipathic DNA polymer (40-nucleotide polycytidine)	HSV	Human cervical epithelial (CaSki)	Reduced viral and protein expression	[120]
Amphipathic DNA polymers (REP 9, REP 2015 and REP 9C)	Animal	NIH 3T3	Reduced viral replication	[123]
REP 2006	HBV	DHBV	Reduced viral entry to the host	[126]
REP 2055	HBV	DHBV	blocking release of DHBsAg from the infected hepatocytes	[128]

**Table 6:** Antiviral activity of dendrimers and their mechanisms of action.

Dendrimer Type	Name	Virus	Cell Line	Mechanism of Action	Ref.
Polyanionic	Phenyldicarboxylic acid (BRI6195), Naphthyldisulfonic acid (BRI2923)	HIV-1	MT-4	Inhibit viral binding and replication	[142]
Polyanionic	SPL-2999	HSV-1, HSV-2	Vero	Inhibit late stages of viral replication	[144]
Polyanionic	carbosilane dendrimer G2-STE16	HIV-1	TZM.bl	Inhibit viral entry	[151]
Polyanionic	G2-S16	HIV	PBMC	Inhibit viral replication by blocking gp120/CD4/CCR5 interaction	[155]
Polyanionic	G3-S16 and G2-NF16	HIV-1	HeLa P4.R5MAGI, TZM.bl	Inhibit viral replication by blocking gp120/CD4/CCR5 interaction	[138]
Polycationic	Viologen	HIV-1	MT-4 cells	Block viral entry	[152]

**Table 7:** List of studies on antiviral activity of sialyl-based polymers with their mechanism of action.

Sialyl-Based Polymers	Virus	Cell Line	Mechanism of Action	Ref.
Amide-sialoside protein conjugates	IAV	H1N1, H3N2, H9N2	Binding to the virus and inhibiting replication	[157]
Thiosialoside-modified poly (methyl vinyl ether-alt-maleic anhydride)	IAV	MDCK	inhibiting both early attachment and late release during viral infection	[160]
silyl oligosaccharides modified glycopolymers	IAV	H1N1	Binding with hemagglutinin on viral surface	[161]
Bioinspired brush polymers containing α-2,6-linked sialic acid	IAV	H1N1	binding hemagglutination on viral surface	[163]

## Data Availability

Not applicable.

## Abbreviations

CXCR4	chemokine receptor type 4
ERK	extracellular signal-regulated kinase
gB	glycoprotein B
H1N1	novel influenza A virus
HBV	hepatitis B virus
HCoV-OC43	human coronavirus OC43
HCV	hepatitis C virus
HDV	hepatitis delta virus
HIV	human immunodeficiency virus
hMPV	human metapneumovirus
HPV	human papillomavirus
HRV	human rhinovirus
HSV-1	herpes simplex virus Type 1
HSV-2	herpes simplex virus Type 2
IAV	influenza A virus
IC50	Half maximal inhibitory concentration
NAPs	nucleic acid polymers
PBMC	primary peripheral blood mononuclear cells
PCD	polyanionic carbosilane dendrimers
PG	2,3-O-disulfated-1,1,4-poly-L-guluronic acid
RSV	respiratory virus
SARS-CoV-2	severe acute respiratory syndrome coronavirus 2
